# Nonbrain metastases seen on magnetic resonance imaging during metastatic brain tumor screening

**DOI:** 10.1007/s11604-022-01362-2

**Published:** 2022-11-14

**Authors:** Mio Sakai, Nobuo Kashiwagi, Katsuyuki Nakanishi, Noboru Maeda, Yasuhiro Nakaya, Junichiro Tanaka, Shinichiro Watanabe, Hidenari Hongyo, Yu Tanaka, Sawaka Yamada, Atsushi Kawata, Sou Toda, Koji Takano, Hideyuki Arita, Noriyuki Tomiyama

**Affiliations:** 1 Department of Diagnostic and Interventional Radiology, Osaka International Cancer Institute, 3-1-69, Otemae, Chuo-Ku, Osaka-Shi, Osaka, 541-8567 Japan; 2 Department of Neurosurgery, Osaka International Cancer Institute, 3-1-69, Otemae, Chuo-Ku, Osaka-Shi, Osaka, 541-8567 Japan; 3 Department of Radiology, Osaka University Graduate School of Medicine, 2-2, Yamadaoka, Suita, Osaka 565-0871 Japan

**Keywords:** Metastasis, Cancer, Magnetic resonance imaging, Central nervous system, Head and neck

## Abstract

Although metastases found during head magnetic resonance imaging (MRI) are not limited to metastatic brain tumors, the MRI is a very common method for “brain metastasis screening,” a modality that is being increasingly performed. In this review, we describe MRI findings of nonbrain metastases and discuss ways to avoid missing these lesions. Metastatic cranial bone tumors are among the most common nonbrain metastatic lesions found on head MRI, followed by leptomeningeal carcinomatosis. The other less-frequent metastatic lesions include those in the ventricle/choroid plexus, the pituitary gland and stalk, and the pineal gland. Metastases in the head and neck area, as well as cranial and intracranial lesions, should be carefully evaluated. Furthermore, direct geographical invasion, perineural spread, and double cancers should also be considered. While it is important to recognize these metastatic lesions on MRI, because they may necessitate a change in treatment strategy that could lead to an improvement in prognosis due to early introduction of therapy, nonbrain lesions should also be given greater attention, given the increasing survival of patients with cancer and advances in MRI technology, such as contrast-enhanced-3D T1-weighted imaging.

## Introduction

Head magnetic resonance imaging (MRI) is being increasingly performed and routinely used for “brain metastasis screening.” For example, in patients with lung cancer, melanoma, and advanced breast cancer, which are most common primary cancers associated with brain metastases, initial screening and surveillance MRIs are performed to detect brain metastases, in accordance with consensus guidelines [1–4]. However, metastases found on head MRI are not limited to brain tumors and approximately 20% of metastases detected on “brain MRI” are nonbrain lesions [5]. Further, apart from limited metastases, direct geographical invasion, perineural spread (PNS), and double cancers also need to be accurately recognized. Owing to both increasing survival of patients with systemic cancer and advances in MRI technology, the number of patients with nonbrain lesions detected on head MRI is increasing. For example, many institutions routinely use contrast-enhanced (CE)-3D T1-weighted imaging (T1WI) as it cannot only delineate small metastatic lesions [6] but also diagnose leptomeningeal carcinomatoses (LMCs) [7]. Further, multiplanar reconstruction (MPR) images obtained in 3D sequences are useful for depicting nonbrain metastatic lesions, and the utilization of fat suppression (FS) in CE-3DT1WI enhances contrast resolution and improves visibility of these lesions, such as bone and soft tissue metastases [8].

Here, we describe the epidemiology of nonbrain metastatic lesions, their MRI findings, and the importance of head MRI checklists for adequately recognizing nonbrain lesions. Given that the pathogenesis of metastasis from solid tumors differs from central nervous system (CNS) invasion by hematological tumors, this review will focus only on metastasis from solid tumors.

## Head MRI checklist and nonbrain metastatic lesions

Nonbrain metastatic lesions in the cranial and intracranial structures include LMC and those seen in the bone (clivus, skull base, and crown of the skull), the ventricle/choroid plexus, the pituitary gland and stalk, and the pineal gland. Among extracranial structures, orbital, cutaneous/subcutaneous tissue, muscle, parotid gland, upper cervical bone, and intramedullary spinal cord metastases (ISCM), and even double cancers in the head and neck, can be observed on head MRI. Additionally, physicians need to be able to recognize PNS and direct geographical invasion. Thus, careful interpretation of head MR images based on a checklist will help accurate identification of nonbrain lesions that may otherwise be missed [9, 10]. Such lesions can be present in the brain, the ventricles, the pituitary gland, the parasellar region, the cerebellopontine angle, the internal auditory canal, vessels, bones (clivus, skull base, and crown of the skull), and extracranial structures. Extracranial structures refer to the head and neck and the spine in all fields of view. Table 1 provides a list of nonbrain metastatic lesions, noteworthy structures for each lesion type, common primary lesions, and primary lesions that should not be missed.

**Table 1:** Nonbrain metastatic lesions, structures, and primary sites

Nonbrain metastatic lesions	Structures	Common primary sites	Don’t-forget primary sites		Comments
**Frequent**					
Bone Mets	Skull and cervical bone	Breast and lung cancer		Prostate and thyroid cancer	30% coexist with brain Met
LMC	Meninges, ventricles, and CNs	Breast and lung cancer		Melanoma	> 70% coexist with brain Met
**Infrequent**					
Pituitary Mets	Pituitary gland and stalk	Breast and lung cancer		Thyroid cancer	
Pineal gland Mets	Pineal grand	Lung and breast cancers		Melanoma	
Ventricle/choroid plexus Mets	Ventricle/choroid plexus	Lung cancer and RCC		Melanoma and gastrointestinal cancer	RCC Mets cause intratumoral hemorrhage and massive edema
Muscle Mets	HN muscles	Breast and lung cancer		Esophageal cancer	Extraocular muscle is the second most site, next to thigh muscle
Orbital and ocular Mets	Eye and orbit	Breast and lung cancer		Skin cancers	Met can cause retinal detachment
Cutaneous/subcutaneous Mets	Cutaneous/subcutaneous tissue	Lung and breast cancer		HN cancers	One-thirds occur in the HN area
Parotid Mets	Parotid gland	Skin SCC and melanoma		HN cancers and RCC	90% are from supraclavicular sites
ISCMs	Cervical spinal cord	Lung and breast cancer		Prostate cancer	ISCM is considered, check lung
Direct invasions and PNS	Skull base and HN	HN cancers		Melanoma	PNS: CNs V and VII are common
Double cancers	Nasopharynx and mesopharynx	N/A		N/A	HN cancers and/or lymph node Met should be noted

*CN* cranial nerve, *DWI* diffusion-weighted images, *HN* head and neck, *ISCM* intramedullary spinal cord metastasis, *Met* metastasis, *MRI* magnetic resonance imaging, *N/A* not applicable, *PNS* perineural spread, *RCC* renal cell carcinoma, *SCC* squamous cell carcinoma

Adapted from [9–11, 16, 20, 24, 26–28, 30, 31, 33–36, 39, 41–43, 45–48, 50–56]

## Frequent nonbrain metastases

Among nonbrain metastases, skull metastases are the most common, followed by LMC, and together with brain metastases, they account for > 95% of all cranial metastases [5].

### Skull metastases

Skull metastases are the most common nonbrain metastatic tumors. They are often asymptomatic and are incidentally detected on head MRI. Major symptoms, if present, include headache and nausea in patients with dural infiltration or cranial nerve (CN) afflictions in patients with skull base metastases. Skull metastases are generally manageable; however, early diagnosis is crucial for effective treatment [11].

The incidence of skull metastasis in patients with breast, lung, and prostate cancers is estimated to be > 20% [11, 12], and the most common primary cancers are breast (> 50%), followed by lung and prostate cancer [11]. Further, as approximately 30% of patients with skull metastases also develop brain metastases [11], the latter should be carefully assessed when skull metastasis is detected. Additionally, as 10% of skull metastases are not accompanied by bone metastases at other sites [13], this finding is not a reason for excluding cranial metastases during diagnosis.

#### Key MRI findings

When interpreting head MR images of patients with systemic cancer, especially of the breast, lung, or prostate, attention should be paid to the bones (clivus, skull base, crown of skull, and cervical bones) (Table 1), and CE-3DT1WI with FS represents a particularly useful multiplanar modality that can facilitate identification of metastases on the top and the base of the skull.

**Fig. 1:**
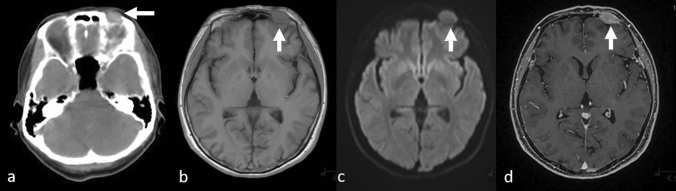
Localized osteolytic skull metastases in a patient with breast cancer. Computed tomography **a** shows osteolytic bone metastasis in the frontal bone (arrow); T1-weighted image **b** shows the tumor as a “gray” mass (arrow), while a diffusion-weighted image **c** shows it as a hyperintense area (arrow) and contrast-enhanced 3D fat-suppressed T1-weighted image **d** shows it as a homogeneously enhanced mass (arrow)

**Fig. 2:**
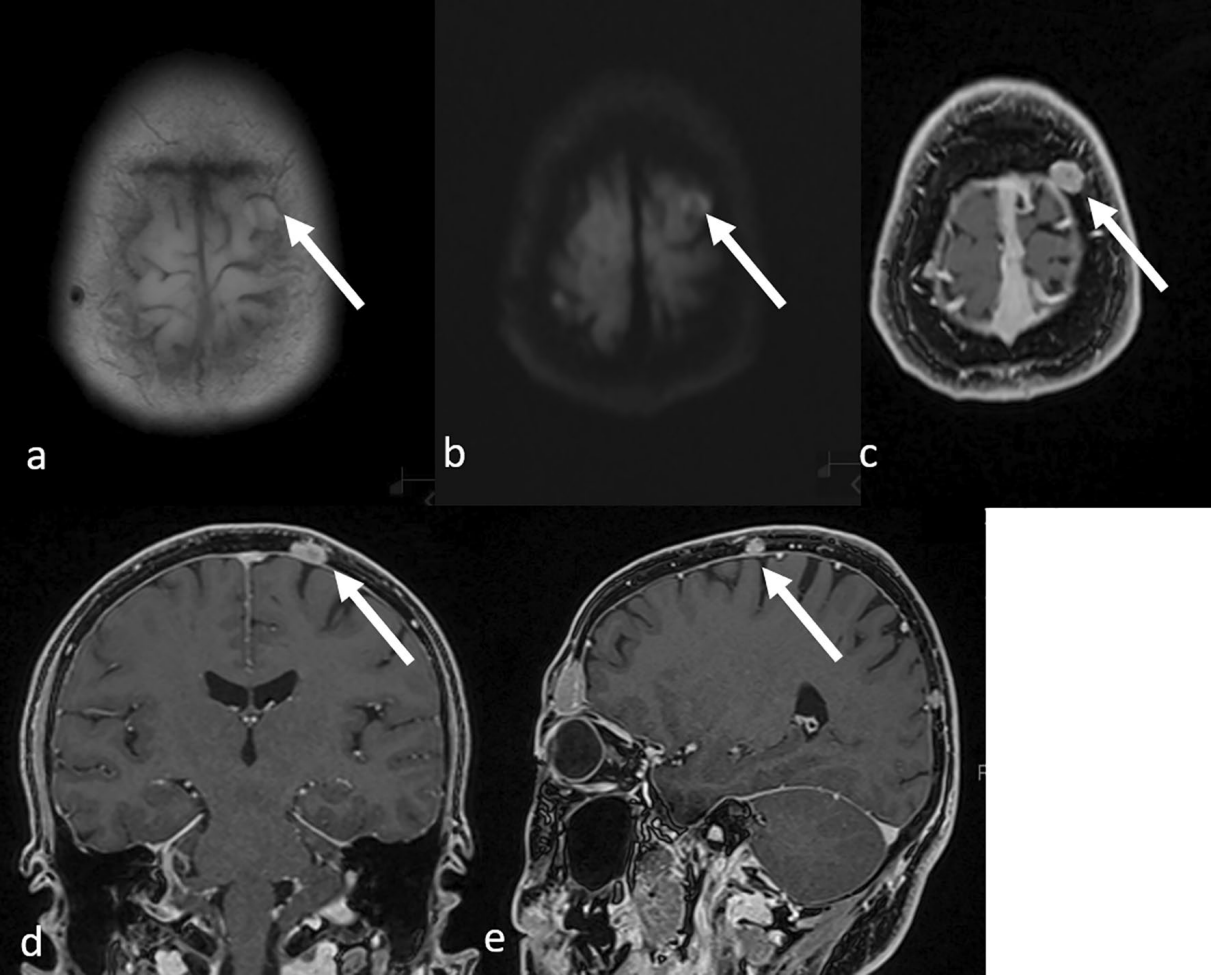
Osteolytic metastases on top of the left parietal bone in a patient with breast cancer. T1-weighted **a** and diffusion-weighted **b** images do not clearly show the tumor (arrows), whereas contrast-enhanced 3D fat-suppressed T1-weighted images with multiplanar reconstruction (MPR) in the axial **c**, coronal **d**, and sagittal **e** views clearly show the lesion (arrows)

**Fig. 3:**
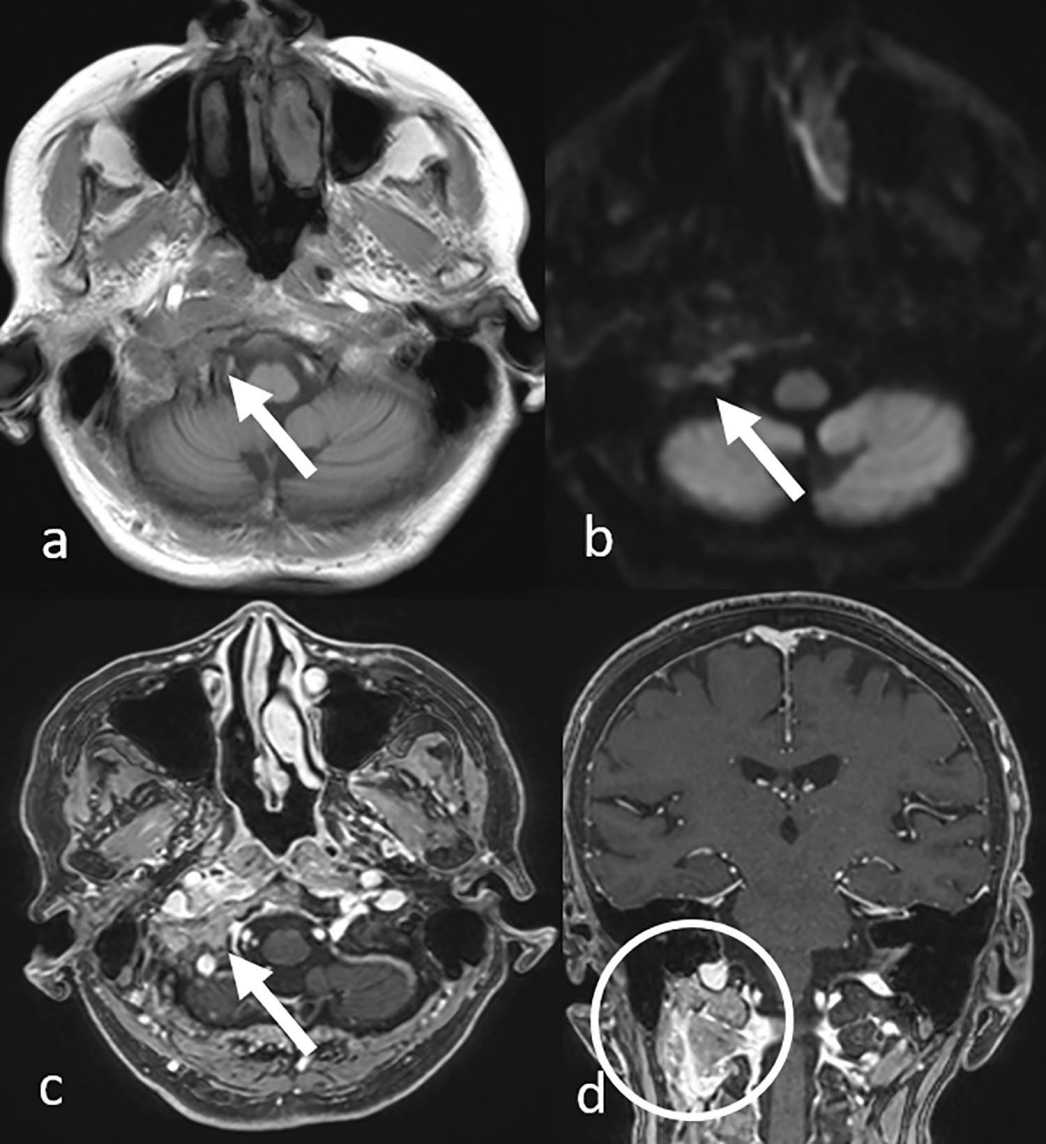
Osteolytic skull base metastasis in a patient with lung adenocarcinoma. T1-weighted **a** and diffusion-weighted **b** images show part of the tumor (arrows). Contrast-enhanced 3D fat-suppressed T1-weighted images with multiplanar reconstruction in the axial **c** and coronal **d** views show the tumor invading extracranial structures (c, arrow and d, circle)

**Fig. 4:**
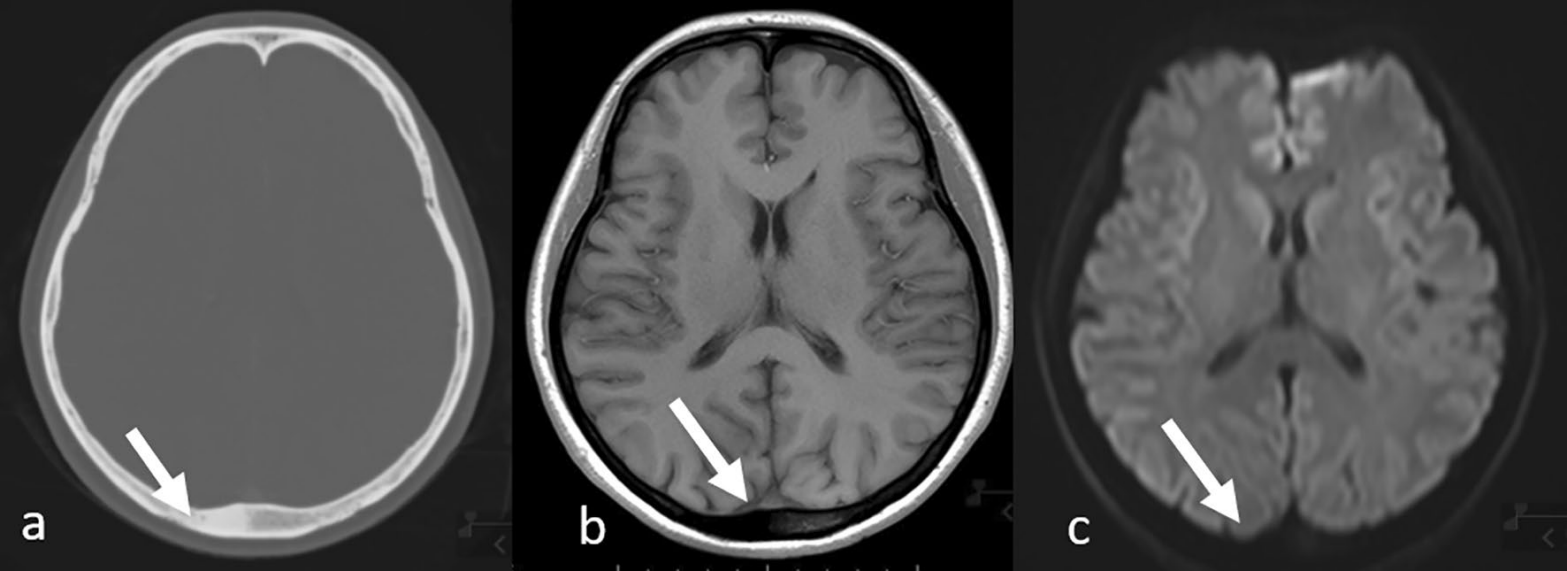
Osteosclerotic skull metastasis in a patient with lung adenocarcinoma. Computed tomography **a** clearly shows the lesion but it is not clearly visible on MRI: T1-weighted **b** and diffusion-weighted **c** images (arrows). *MRI* magnetic resonance imaging

Skull metastases can be localized or diffused, and the most common are those localized in the cranial crown or the diffuse lesions in the skull base [11]. On T1WI, a “gray” lesion replacing the “white” normal bone marrow adipose tissues is indicative of bone metastases (Fig. 1 a, b) [5, 11]; however, the T2WI signal is variable [11]. Diffusion-weighted imaging is useful for detecting osteolytic lesions, such as the majority of breast and non-small cell lung cancer metastases that show high signal intensity (Fig. 1c) [5, 14]. On CE-T1WI, FS is desirable because the signal from a contrast-enhanced lesion might be equivalent to that of the background marrow adipose tissue (Fig. 1d). CE-3DT1WI is useful to detect the dural and CN infiltration of bone metastases as it permits greater spatial resolution than conventional CE-T1WI. In the presence of dural invasion, a thickened dura arachnoid with contrast enhancement is shown [5]. In the presence of CN infiltration, CE-3DT1WI shows a contrast-enhancing effect on the CNs [5]. Multiplanar observation, especially MPR on CE-3DT1WI with FS, is useful for detecting metastases on the top and the base of the skull (Figs. 2, 3). MRI is useful for detecting osteolytic lesions but should be cautiously observed for osteosclerotic lesions. CT shows the superior delineation of osteosclerotic lesions (Fig. 4).

### LMC

LMC is clinically detected in 5–8% of patients with cancer [15], and breast cancer is the most common primary solid tumor, accounting for 27–50% of all patients with LMC, followed by lung cancer (22–36%) and melanoma (12%) [16].

The clinical signs of LMC are predominantly caused by focal or global involvement of the brain hemispheres (15%), the CNs (35%), and/or the spinal cord and nerve roots (60%); nonetheless, multifocal involvement and signs of intracranial hypertension (26%) are also frequently seen [16]. Notably, prognosis is poor with a median survival of 1–4 months [17], but LMC may sometimes be detected incidentally on head MRI before a patient becomes symptomatic [16, 18]. Outcomes continue to improve with the development of systemic/intrathecal options; hence, early diagnosis is desirable [17].

#### Key MRI findings

When LMC is clinically suspected, the two main diagnostic tools are MRI and cerebrospinal fluid (CSF) analysis [16, 18]. As MRI is a powerful diagnostic tool for LMC, it is important to examine the meninges, the ventricles, the cerebellopontine angle, and the internal auditory canals for key findings (Table 1). CE-MRI of the brain and spinal cord has a specificity of 70–80% for LMC, which is superior or equal to that of repeated CSF cytology [16, 18]. Nevertheless, as normal MRI findings cannot be used to exclude LMC, CSF cytology and MRI are performed together, along with evaluation of clinical findings, for accurate diagnosis [18].

**Fig. 5:**
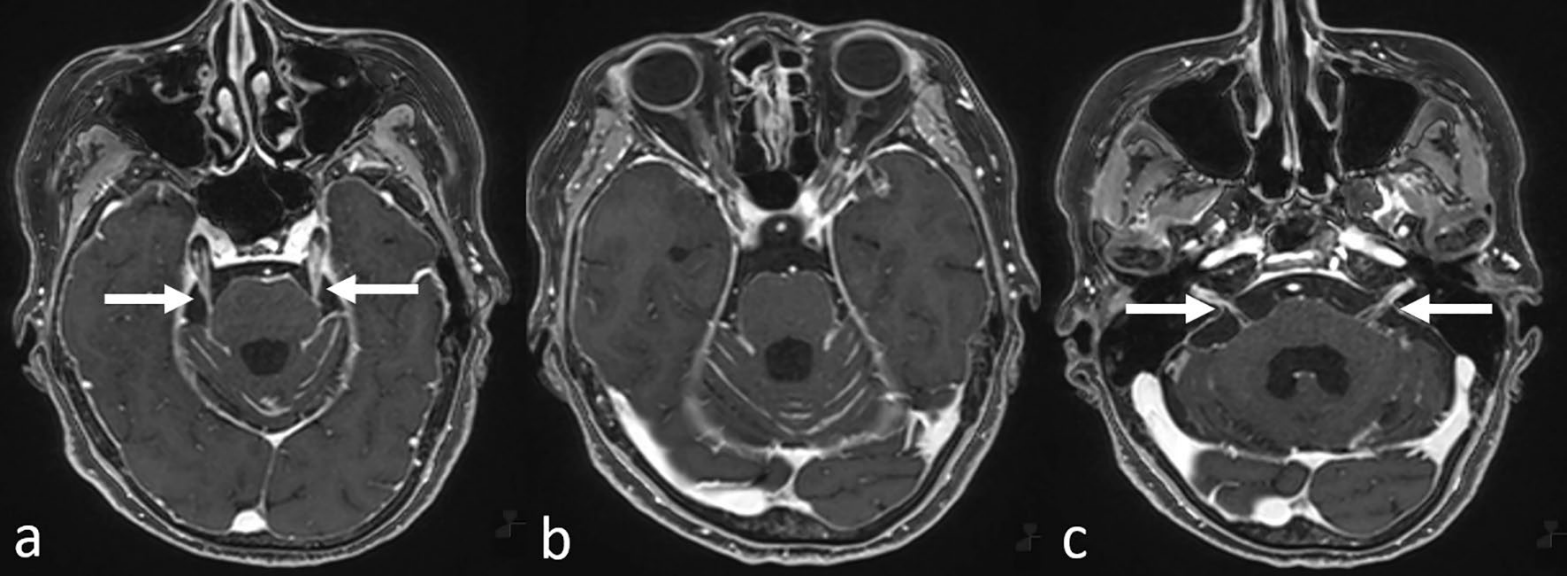
Leptomeningeal carcinomatosis in a patient with gastric cancer. Contrast-enhanced 3D fat-suppressed T1-weighted images show enhancement in the bilateral cranial nerve V (a, arrows), brain stem and cerebellar meninges (**a**–**c**), and bilateral internal auditory canal corresponding to cranial nerves VII/VIII (c, arrows)

CE-T1WI and fluid-attenuated inversion recovery (FLAIR) sequences have the highest sensitivity for detecting LMC [19]. The MRI findings compatible with LMC diagnosis are smooth or focal enhancement of the meninges, subarachnoid or intraventricular nodules, and focal enhancement in the ependyma, CNs, and nerve roots on CE-T1WI (Fig. 5) [5, 16, 19]. Specifically, neoplastic LMC predominantly involves the cerebellum and the occipital lobes (Fig. 5 a, b), CN VII/VIII (Fig. 5c), and ependymal lining of the lateral ventricles [20], and CE-3DT1WI adequately delineates these structures as it permits greater spatial resolution compared to conventional CE-T1WI [7].

**Fig. 6:**
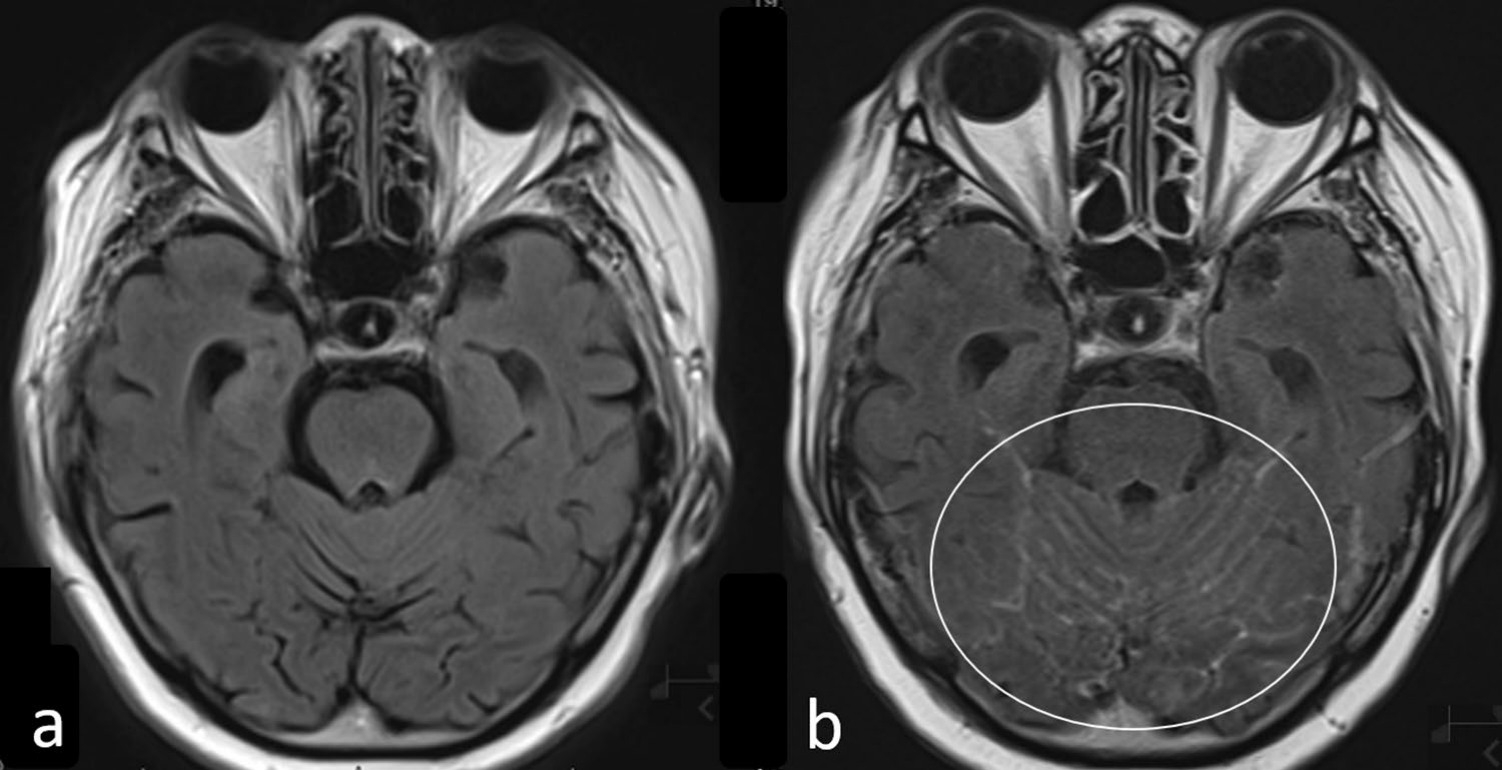
Leptomeningeal carcinomatosis in a patient with breast cancer. Fluid-attenuated inversion recovery image without contrast enhancement **a** shows no abnormal signal while post-contrast fluid-attenuated inversion recovery image **b** shows meningeal enhancement (circle)

**Fig. 7:**
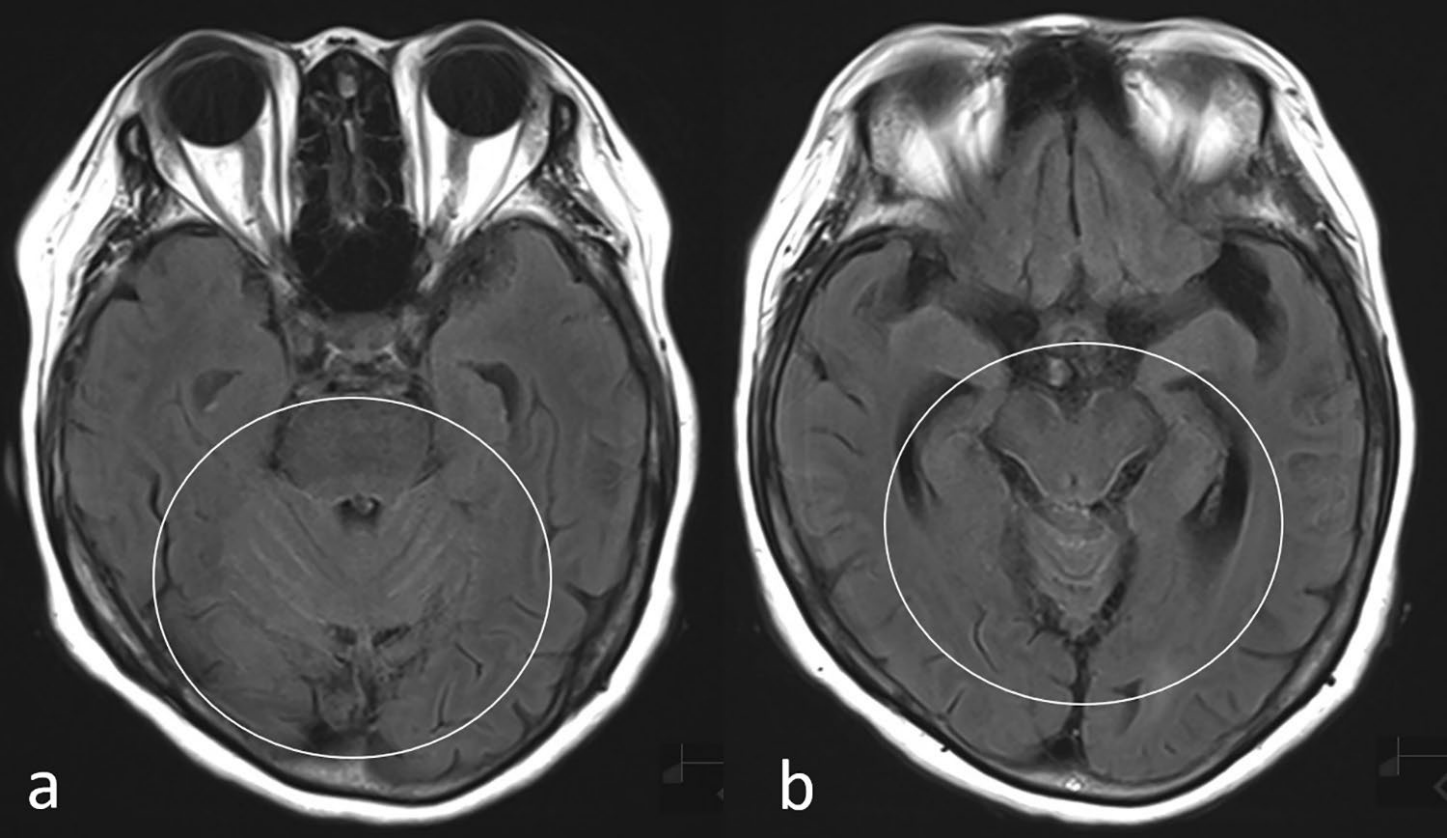
Leptomeningeal carcinomatosis in a patient with cholangiocarcinoma. Noncontrast-enhanced fluid-attenuated inversion recovery images show hyper-intensity in the cerebellar sulci and brain stem surface (**a** and **b**, circles)

Hydrocephalus is also commonly seen on imaging in patients with LMC, and in patients with systemic cancer, LMC should be suspected when enlarged ventricles are seen. However, it may be difficult to detect hydrocephalus, especially in elderly patients, and a comparison of current and previous MR images is useful in such patients. CE-FLAIR images are equal or superior to CE-T1WIs in delineating meningeal enhancement (Fig. 6) [21] with non-CE-FLAIR images showing hyperintense sulci or cisterns in some patients with LMC (Fig. 7). Thus, hyperintense sulci or cisterns on non-CE-FLAIR images in patients with cancer should prompt a strong suspicion of LMC, and CE images should be obtained [21]. Further, as non-CE and CE-FLAIR and CE-3DT1WI are complementary, it is helpful to acquire both sets of sequences [7].

At diagnosis, brain and extracranial metastases are seen in > 70% and > 80% of LMC patients, respectively [22]; hence, when the above signs are seen on head MRI and LMC is suspected, careful interpretation is required to identify both brain and nonbrain metastases [22].

## Infrequent nonbrain metastases in the cranial region

Although metastases in other intracranial regions, the pituitary gland and stalk, the pineal gland, and the ventricle/choroid plexus metastases are uncommon with a combined incidence of < 5% [5], improvements in MRI technology that enhance delineation of target structures have led to a more frequent diagnosis of these rather rare metastases in daily practice, albeit as incidental findings.

To avoid missing these infrequent metastases, it is useful to interpret MRI scans according to the structures provided in the checklist (Table 1). For detecting metastases in the pituitary gland and stalk and the pineal gland, the parasellar and pineal regions should be checked, for which sagittal images, especially with CE-3DT1WI, are especially useful. For detecting ventricle/choroid plexus metastases, the ventricles, especially the choroid plexus and the ventricular wall, should be checked. As MRI findings are nonspecific in many of these infrequent nonbrain metastases, they should be considered as differential diagnoses, especially in patients with systemic cancers.

### Pituitary metastases

**Fig. 8:**
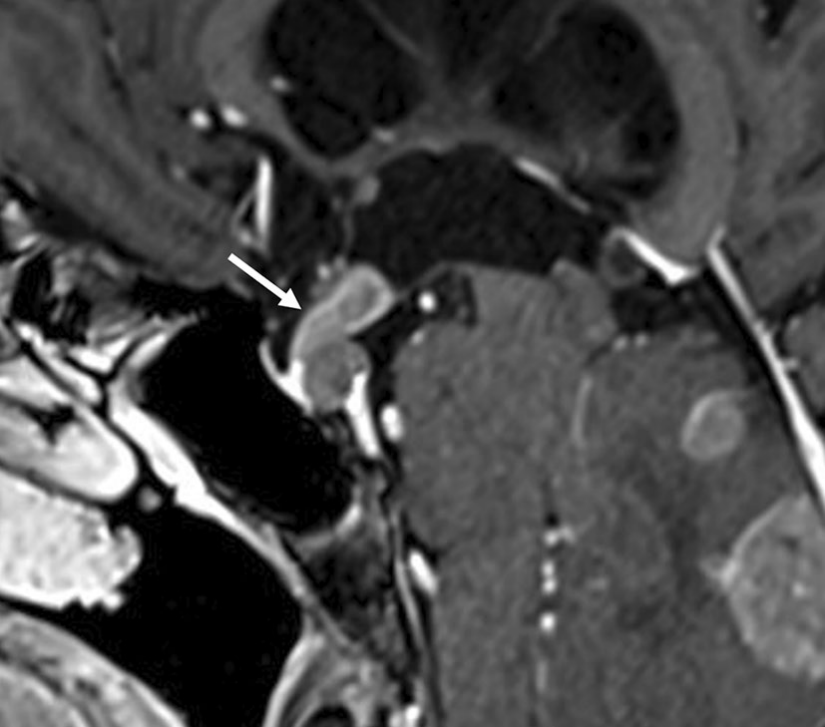
Pituitary metastasis in a patient with breast cancer. A sagittal contrast-enhanced 3D fat-suppressed T1-weighted image shows a dumbbell-shaped pituitary and stalk mass (arrow). Cerebellar metastases and a narrowed fourth ventricle are also visible

Several pituitary metastases are detected incidentally by MRI [23] and they are clearly visible in the sagittal view, especially on CE-3DT1WI (Fig. 8). Approximately 0.14–3.6% of intracranial metastases develop in the pituitary [24], and nearly 1.8% of all surgically resected pituitary masses are metastases [24]. Breast cancer is the most common primary lesion with an incidence rate that is 9.3 times higher than that of other types of cancers, followed by lung and thyroid cancers [24]. Pituitary metastases may be located on the anterior and posterior lobes or the stalk [24], and when symptomatic, the most common manifestations are diabetes insipidus, panhypopituitarism, and vision disturbances [24].

#### Key MRI findings

The most common finding is the presence of an infiltrating enhanced pituitary and/or stalk mass that is dumbbell-shaped with loss of the posterior pituitary “bright spot” on T1WI (Fig. 8) [23]. Macroadenoma is the major differential diagnosis of pituitary metastasis, but it is rarely present with diabetes insipidus. Other differential diagnoses include lymphocytic hypophysitis and IgG4-related hypophysitis, because they resemble pituitary metastasis on imaging studies [25] and often, cause diabetes insipidus. Importantly, MRI cannot distinguish between pituitary metastases and other lesions, and in patients with a known systemic cancer, especially breast cancer, rapid growth of a pituitary mass with onset of clinical diabetes insipidus is highly suggestive, but certainly not diagnostic, of metastasis [23, 24].

### Pineal gland metastases

**Fig. 9:**
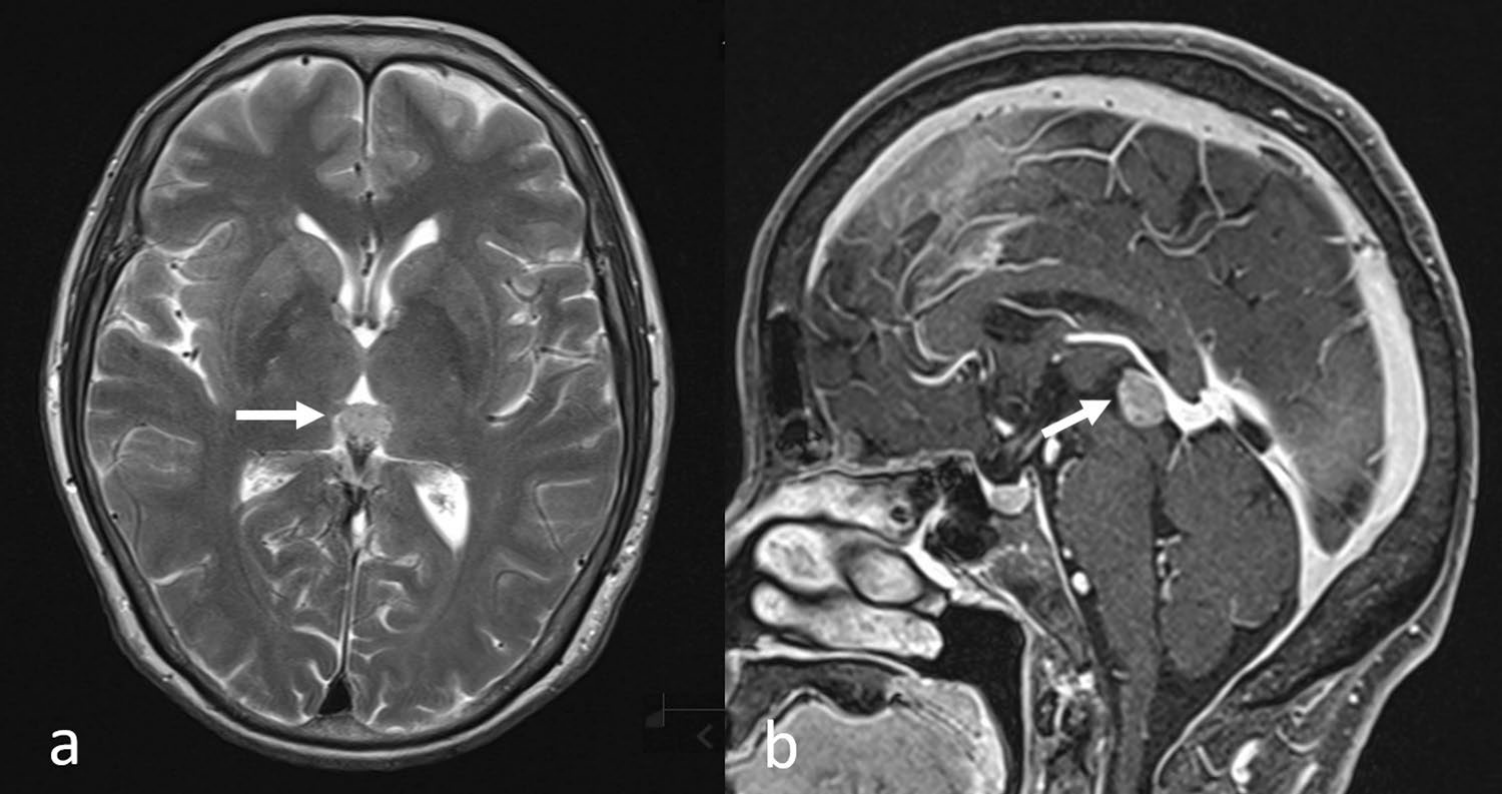
Pineal gland metastasis in a patient with lung adenocarcinoma. T2-weighted image **a** shows a mild hyperintense mass in the pineal area (arrow); sagittal contrast-enhanced 3D fat suppressed T1-weighted image **b** shows the pineal tumor with homogeneous enhancement (arrow)

Consistent with pituitary metastases, metastases to the pineal gland are incidentally discovered upon MRI [23, 26]. Approximately 2.7% of pineal tumors are metastatic and predominantly originate from lung carcinomas, followed by breast cancer [26, 27]. The main route used by extracranial malignant tumors to reach the pineal region remains hematogenous dissemination [27] and their incidence appears to be increasing, probably due to better visualization by MRI. These tumors are clearly seen in the sagittal view, especially on CE-3DT1WI (Fig. 9), and as MRI does not distinguish pineal metastases from other pineal lesions [26, 27], metastasis should always be included as a differential diagnosis of pineal lesions, especially in patients with lung and breast cancers.

### Ventricle/choroid plexus metastases

Ventricle/choroid plexus metastases account for 0.9–4.6% of all intracranial metastases [28]. They usually appear as a single lesion, and the lateral ventricle is the most common site for metastatic spread, followed by the third ventricle. Only 0.4% of all ventricular metastases are located in the fourth ventricle [28].

The most likely primary sources are renal cell carcinoma (RCC) and lung carcinoma [28, 29]. RCC is noteworthy for its propensity to produce a solitary metastasis that can be seen even 50 years after detection of the primary lesion and for its imaging appearance that mimics that of an intraventricular meningioma [28]. Compared to metastasis from other primary cancers, RCC metastasis to the choroid plexus has a greater propensity for intratumoral hemorrhage and massive surrounding edema [30, 31].

#### Key MRI findings

**Fig. 10:**
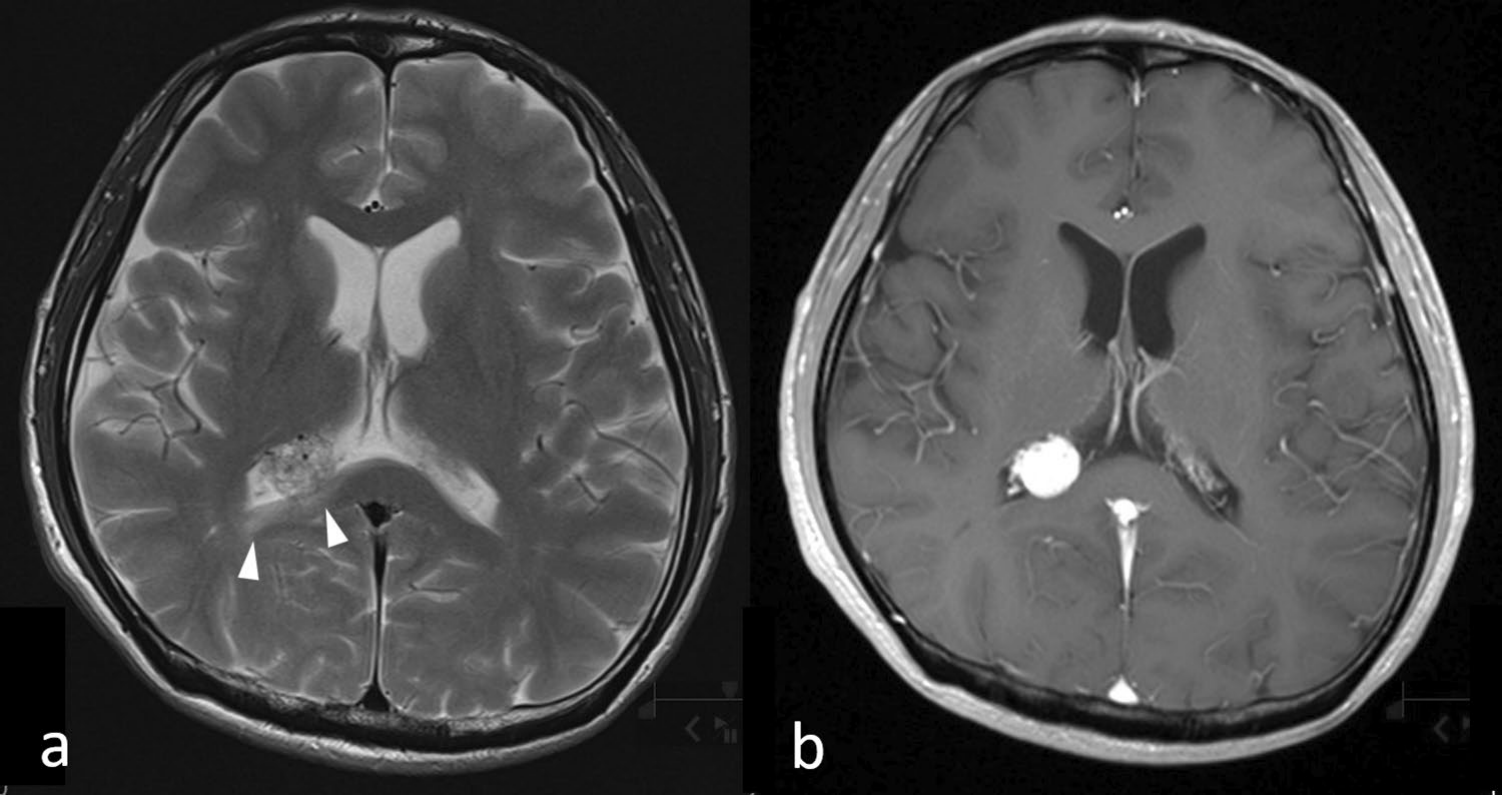
Choroid plexus metastasis in a patient with renal cell carcinoma. T2-weighted image **a** shows a moderate hyperintense tumor in the right lateral ventricle with surrounding hyperintense area (arrow heads) corresponding to edema in the right part of the splenium of the corpus callosum. A contrast-enhanced T1-weighted image **b** shows the tumor with homogeneous intense enhancement

Most nonhemorrhagic choroid plexus metastases are hypointense on T1WI and hyperintense on T2WI/FLAIR (Fig. 10a) [28]. The presence of intense enhancement, either homogeneous or heterogeneous, on the CE-T1WI is typical (Fig. 10b) [32]. With larger lesions, peritumoral edema or invasion into the adjacent brain parenchyma may occur (Fig. 10a) [28]. Differential diagnoses for choroid plexus metastases include intraventricular meningioma and subependymoma, and differentiating among them on imaging may be challenging. Thus, in patients with a known systemic cancer such as RCC, the differential diagnosis for a choroid plexus mass should always include metastasis [5, 28].

## Extracranial metastases, direct invasions, PNS, and double cancers

Head and neck metastases, such as in soft tissue, the parotid gland, the orbit (extraocular and intraocular), or the cervical spine and cord, can be incidentally detected on head MRI. Furthermore, apart from metastases, PNS and direct geographical invasion should be assessed when interpreting head MR images. Additionally, as double cancers in the head and neck area can be incidentally found on head MRI, careful interpretation using checklists is needed not only for the cranial and skull areas but also the head and neck region (Table 1).

### Muscle metastases

The prevalence of muscle metastases has been reported to vary from 0.03 to 5.6% in an autopsy series and from 1.2 to 1.8% in a radiological series [33]. Almost one-fifth of all muscle metastases are located in the head and neck areas [33]. The extraocular muscle is the second most frequent site of metastases, next to the thigh muscle, and extraocular muscle metastases account for 15% of all muscle metastases [33–36]. Breast and lung cancers are the most common primary malignancies [33–36], and although rare, muscle metastasis from esophageal cancer is more likely to be located in the head and neck region [33].

#### Key MRI findings

**Fig. 11:**
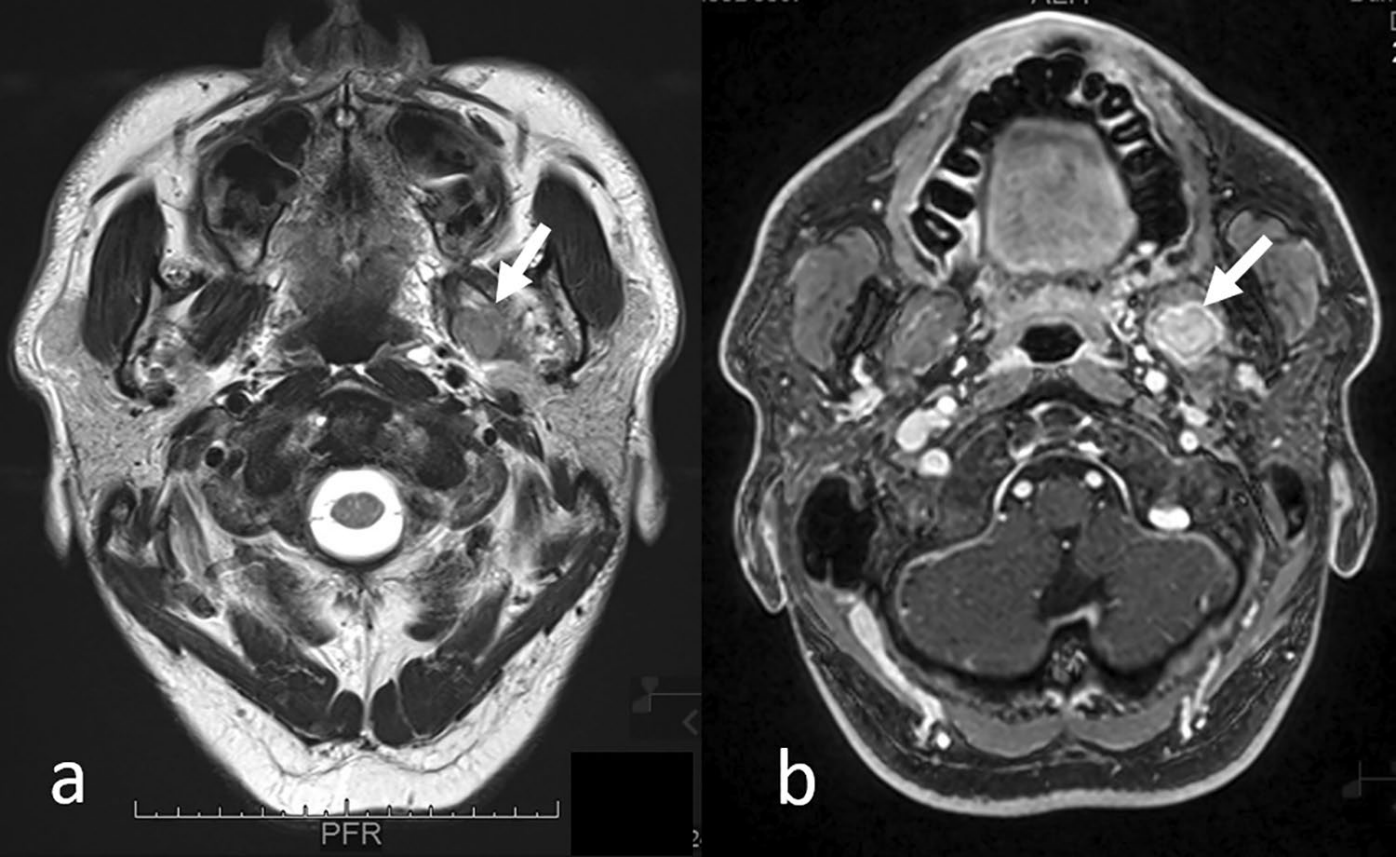
Well-defined left pterygoid muscle metastasis in a patient with a lung adenocarcinoma. T2-weighted image **a** shows the tumor as slightly hyperintense compared to the contralateral pterygoid muscle (arrow). Contrast-enhanced 3D fat-suppressed T1-weighted image **b** shows homogeneous, well-defined contrast enhancement of the tumor (arrow)

**Fig. 12:**
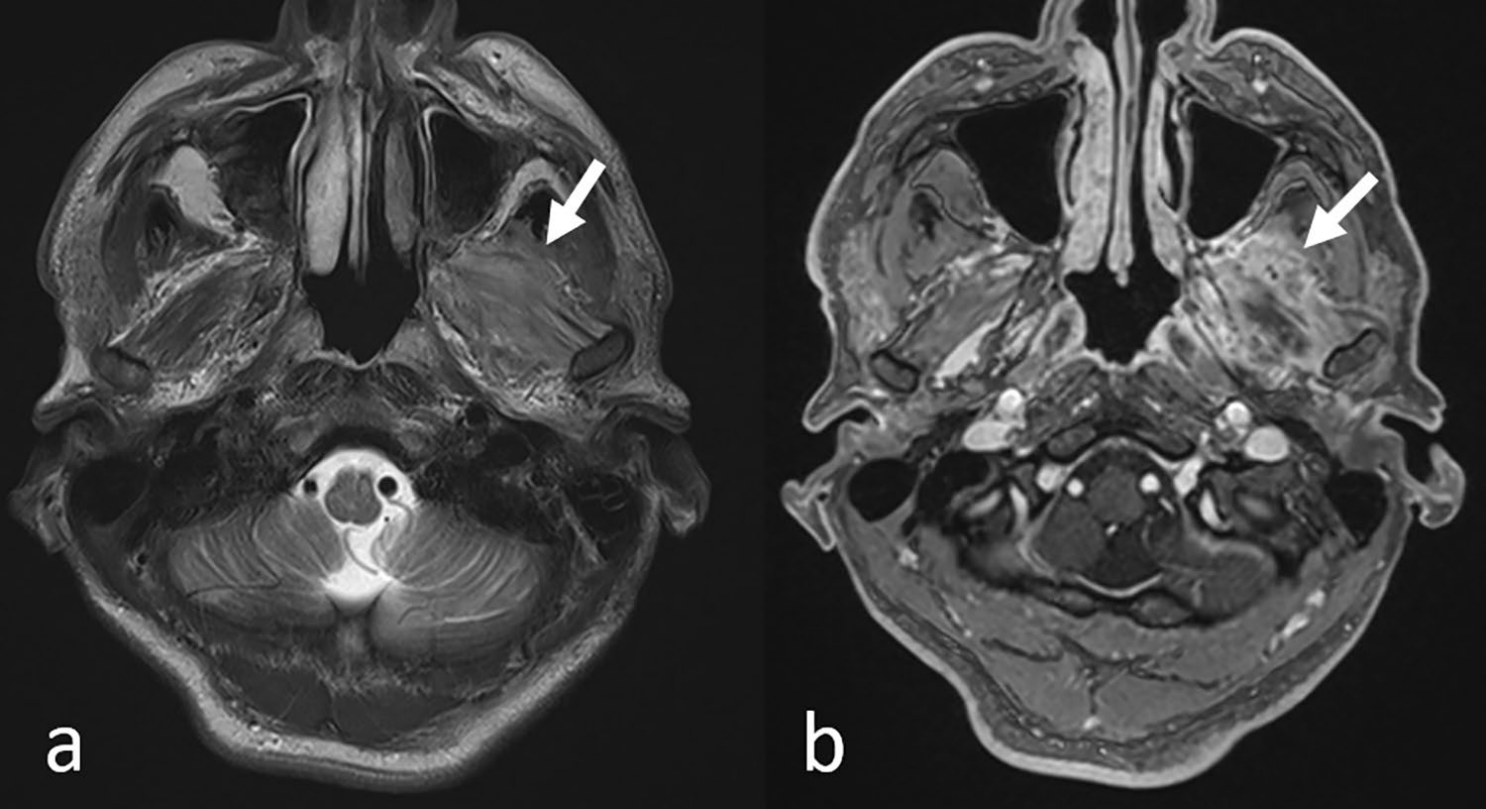
Diffuse left pterygoid muscle metastasis in a patient with esophageal cancer. T2-weighted image **a** shows that the swollen left pterygoid muscle has mildly higher intensity compared to the contralateral pterygoid muscle (arrow). Contrast-enhanced 3D fat-suppressed T1-weighted image **b** shows heterogeneous enhancement with an irregular margin of the swollen left pterygoid muscle (arrow)

**Fig. 13:**
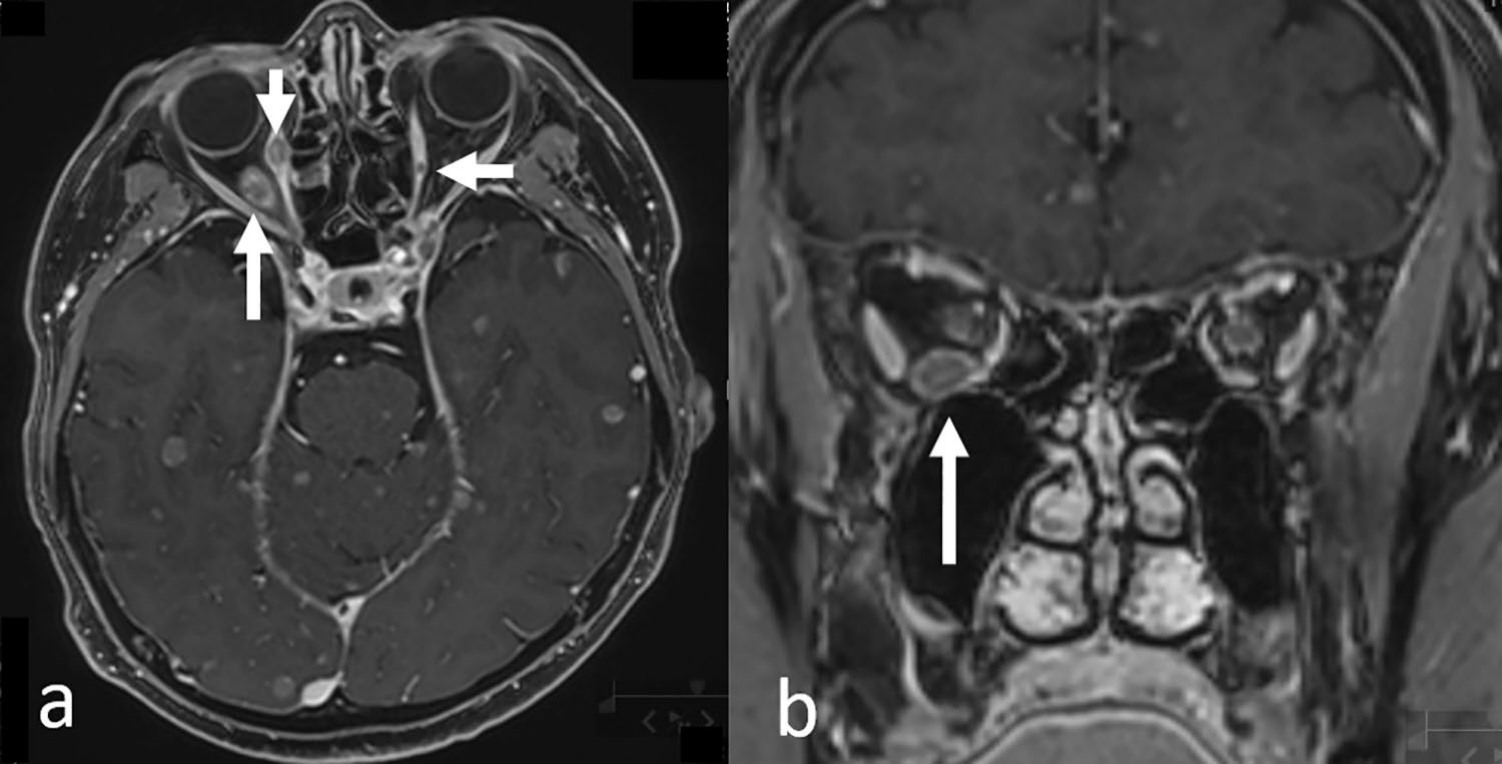
Multiple well-defined extraocular muscle metastases in a patient with small cell carcinoma of the prostate. Axial **a** and coronal **b** reconstructed contrast-enhanced 3D fat-suppressed T1-weighted images show multiple well-defined nodular tumors as less contrast-enhanced masses (arrows) in the extraocular muscles

**Fig. 14:**
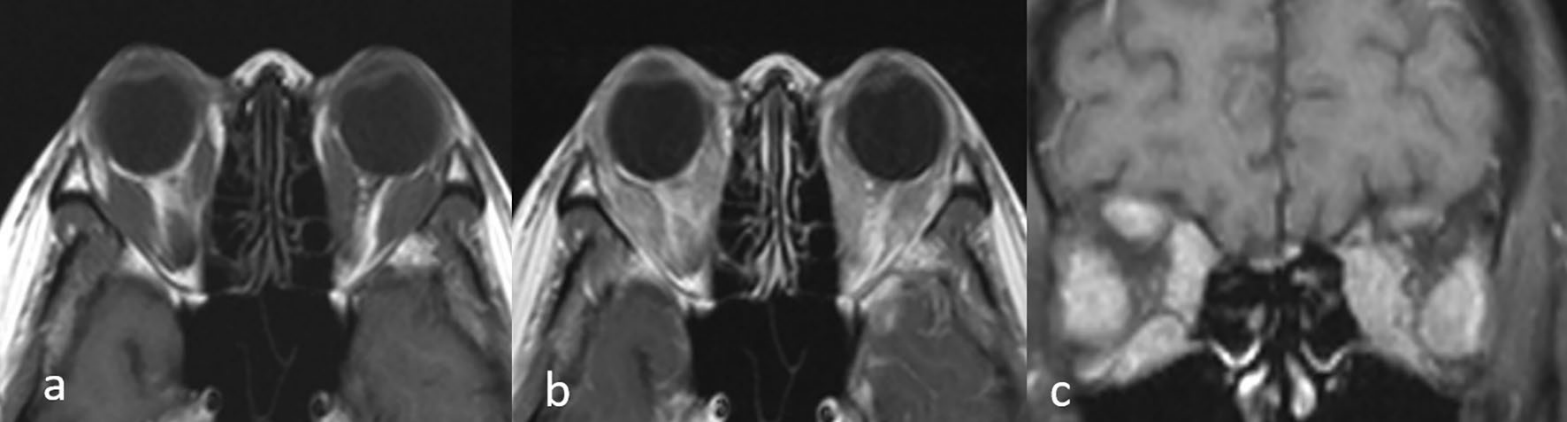
Multiple diffuse extraocular muscle metastases in a patient with solid tubular carcinoma of the breast. T1-weighted image **a** shows diffuse swelling of extraocular muscles. Contrast-enhanced T1-weighted image **b** and coronal contrast-enhanced T1-weighted image with fat suppression **c** show diffuse enhancement of the swollen extraocular muscles

Generally, tumors can be focal (Fig. 11) or diffusively infiltrative (Fig. 12), and no specific MRI features of muscle metastases have been described [33]. Calcifications and/or hemorrhage can be seen but are rarely observed [33]. Most metastases show an intermediate signal on T2WI and have a ring-like, nodular, homogeneous, or heterogeneous enhancement on CE-T1WI (Figs. 11, 12) [33, 34, 37]. Further, while well-defined tumors are specific for extraocular muscle metastases (Fig. 13), infiltrative tumors are difficult to differentiate from lymphoma or inflammatory diseases (Fig. 14) [37]. Thus, differentiating extraocular muscle metastases, especially diffuse infiltrative ones, from nonneoplastic infiltrative processes, such as an orbital pseudo tumor, thyroid ophthalmopathy, or granulomatous disease, poses a diagnostic challenge [35].

### Orbital and ocular metastases

Ocular metastases are significantly more common than orbital metastases, outnumber orbital metastases by eight to one [35, 38, 39], and preferentially affect the vascularly rich choroid membrane [40, 41]. The most common primary lesion is breast cancer (37–41%), followed by lung cancer (21–26%) [41, 42]. Gradual visual loss is the most common clinical finding [43], and systemic cancer is not known in 10% of patients with ocular metastases [41]. Choroidal metastases on T2WI appear as hypointense intraocular lesions that are adjacent to the sclera [43]; however, in some cases, the tumors are associated with a large amount of subretinal fluid, which is unusual for their size. Such patients may present with complete retinal detachment, which can be seen on both CT and MRI [43]. Thus, systemic cancer should be considered as one of the causative diseases of retinal detachment seen on imaging [41].

Orbital metastases represent 1–13% of all orbital tumors [35, 36, 38, 39], and several studies have reported that the most frequent primary cancer site is the breast (21.6–58.5%), followed by the lung (5–12%) and the skin (3–20%) [39]. Further, while breast cancer metastasis has a tendency to localize to the orbital fat pad and muscle, melanomas have a strong preference for muscles [35]. Proptosis and motility disturbances are among the most common presenting signs [34, 35], and clinical symptoms generally manifest rapidly, with progression occurring over weeks to months [34, 35].

MRI, especially CE-3DT1WI with FS, is useful for detecting orbital metastases, which can be an incidental MRI finding [35]. In fact, 19–32% of patients with orbital metastases do not have a known primary tumor at the time of orbital involvement [36], and one-third of patients with orbital breast metastases also have brain metastases [38]. Fat metastases demonstrate diffuse enhancement [35] and MRI findings of extraocular muscle metastases are discussed in the muscle metastases section (Figs. 13, 14). The bony orbit should be routinely examined when evaluating orbital soft tissue metastases, and prostate cancer has tendency to progress into bone metastases [35]. All relevant MRI findings have been discussed in the skull metastases section.

### Cutaneous/subcutaneous metastases

Cutaneous/subcutaneous metastases occur infrequently and account for 0.5–9% of all patients with cancer [44–46]. One-third of cutaneous/subcutaneous metastases occur in the head and neck, with their incidence is almost equal to that of occurring in the chest [47]. Lung, breast, and head and neck cancers are the most common primary cancers that lead to the development of cutaneous/subcutaneous metastasis in the head and neck [45–48].

These metastases are often detected based on a patient complaint or visual examination; however, 70% of cutaneous/subcutaneous metastases are asymptomatic and they are often found only upon imaging, because the presence of hair impedes their recognition [47]. Although a long-time lag is generally observed between diagnosis of primary malignancy and recognition of cutaneous/subcutaneous metastases [45, 47], these metastases can also be the first indication of a clinically silent visceral malignancy [47, 48].

**Fig. 15:**
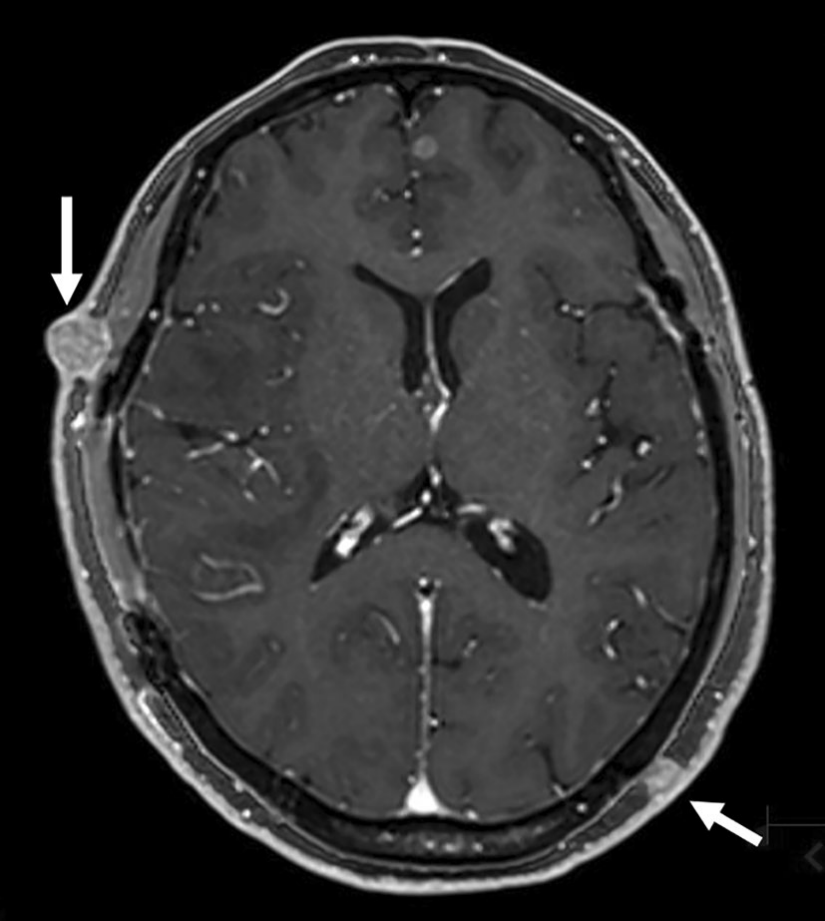
Cutaneous/subcutaneous metastases in a patient with esophageal cancer. A contrast-enhanced 3D fat-suppressed T1-weighted image shows homogeneously enhanced cutaneous/subcutaneous nodules (arrows)

Cutaneous/subcutaneous metastases can present as single or multiple small (< 2 cm) nodules or as an infiltrative nodule [45, 47] with homogeneous or heterogeneous contrast enhancement on MRI (Fig. 15) [45]. Recurrences at surgical sites are usually ill-defined and involve both cutaneous and subcutaneous tissues [45]. Imaging findings are nonspecific and the presence of cutaneous/subcutaneous metastases should be carefully assessed when there is a history of systemic cancer [47]. A biopsy is mandatory for establishing diagnosis and for prompt evaluation of the occult primary malignancy as cutaneous/subcutaneous metastases may often be confused with benign conditions [45].

### Parotid metastases

Approximately 10% of parotid malignancies are metastatic tumors [49] and most occur due to lymphatic spread of head and neck tumors to the parotid lymph node [50, 51]. Metastatic cutaneous squamous cell carcinoma and metastatic melanoma are the most common pathologies that metastasize to the parotid gland, and systemic metastases may also occur from tumors such as RCC [51].

**Fig. 16:**
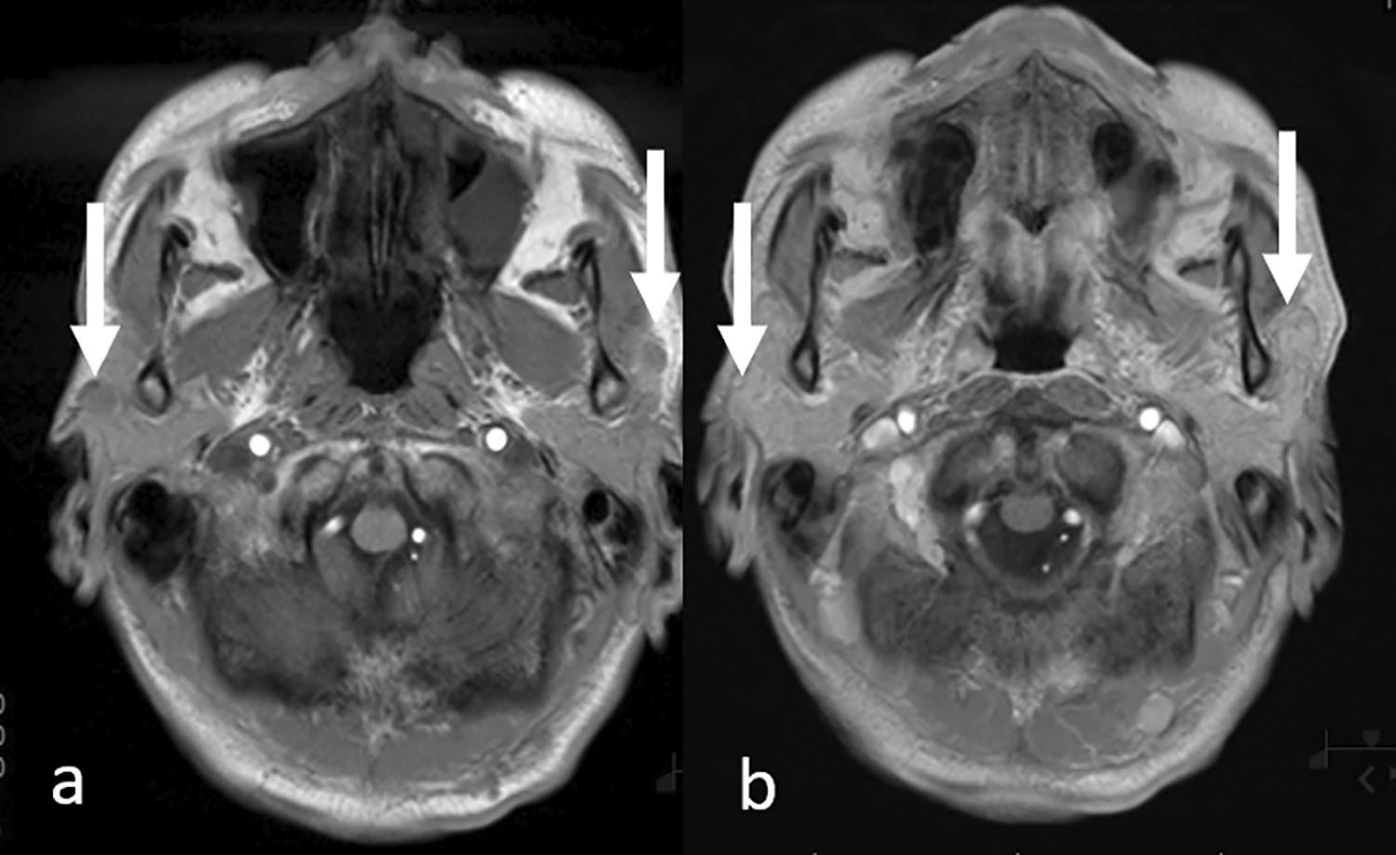
Multiple bilateral parotid metastases in a patient with melanoma. T1-weighted image **a** shows multiple hypointense nodules in the bilateral parotid gland (arrows) corresponding to swollen intraparotid lymph nodes, while a contrast-enhanced T1-weighted image **b** shows a poor contrast between the background and tumor signals (arrows)

On MRI, margins of parotid metastases can be both irregular and well-defined with associated central necrosis [50], and should be differentiated from benign parotid tumors, such as Warthin’s tumor [50]. Notably, the presence of indistinct margins or central necrosis suggests a diagnosis of metastasis, whereas bilateral lesions or an intratumoral cystic component in a discrete location indicates a Warthin’s tumor [50]. As the parotid gland is normally well-enhanced on gadolinium contrast imaging, CE-T1WI may show poor contrast between the tumor and the background, and hence, T1WI without CE, may be optimal (Fig. 16).

### ISCMs

ISCMs can be incidentally detected on head MRI and shows specific findings. ISCMs frequently occur in the cervical spine, and 10% of ISCMs are asymptomatic [52, 53]. Cervical ISCM can be incidentally found on head MRI [52], especially on sagittal views of CE-3DT1WI.

The prevalence of ISCM has been reported to be 2.1% in an autopsy series of patients with cancer [53, 54] and accounts for 1–3% of intramedullary spinal cord neoplasms [55]. With increasing morbidity of cancer and prolonged patient survival, the incidence of ISCM appears to be increasing [52]. Lung and breast cancers are the most common primary site and require special attention [52, 54, 55].

#### Key MRI findings

**Fig. 17:**
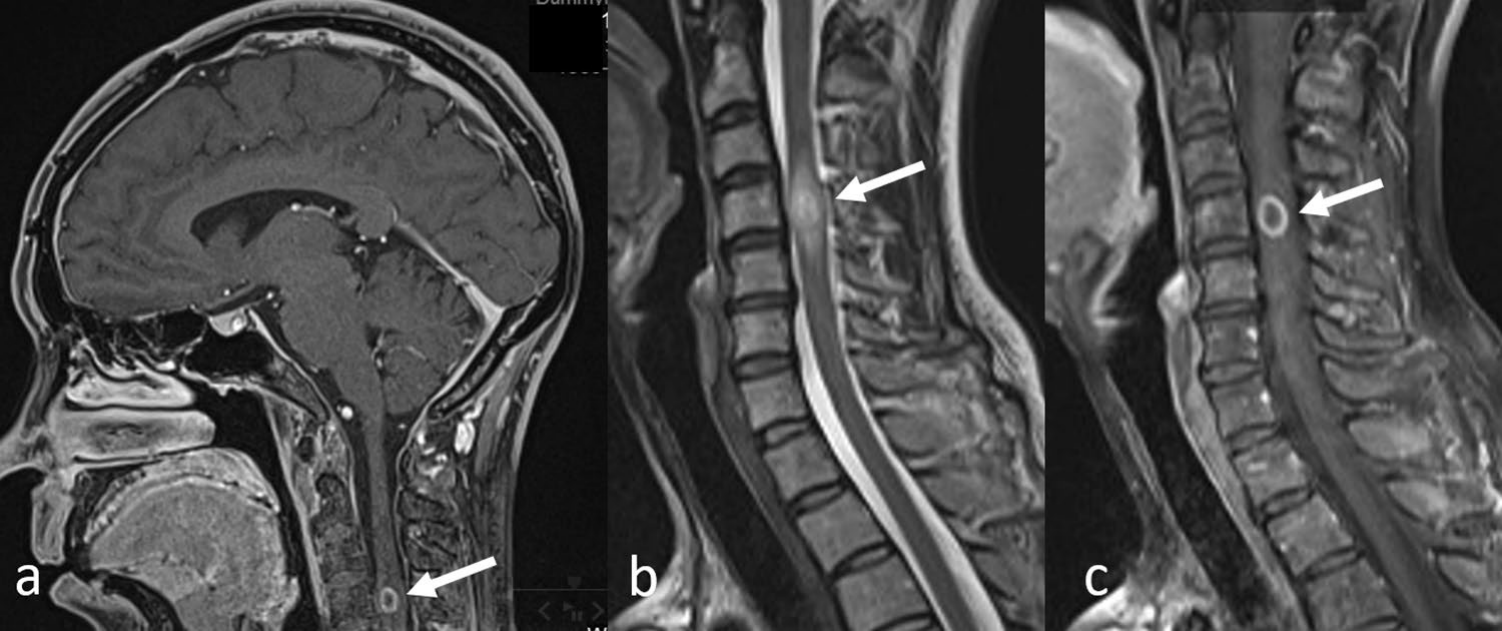
Intramedullary spinal cord metastasis in a patient with lung adenocarcinoma. Contrast-enhanced 3D T1-weighted head MR image in the sagittal view **a** shows a ring-enhanced intramedullary tumor (arrow). Sagittal T2-weighted **b** and contrast-enhanced T1-weighted **c** spine MR images show characteristic findings of extensive edema surrounding the tumor (arrows). *MR* magnetic resonance

Contrast enhancement and extensive tumor-associated edema are typical for ISCMs [53]. On CE-T1WI, almost all ISCMs show an enhancement, which may be both homogeneous and heterogeneous (Fig. 17a). Peripheral edema with T2WI hyper-intensity is extensive and three times larger than the area showing CE (Fig. 17b, c) [53]. The lack of enhancement and presence of cystic change and/or hemorrhage in an intramedullary mass should prompt a search for other etiologies for the spinal cord mass that are more probable differential diagnoses, such as primary cord neoplasms, in which such findings are not uncommon [53].

### Direct invasions from head and neck tumors and PNS

Direct geographical invasion or PNS into the CNS should be evaluated, especially in patients with head and neck cancers, and MRI plays an important role in their diagnosis. As shown in Table 1, careful observation of the skull base and extra cranial structures is required (Table 1).

**Fig. 18:**
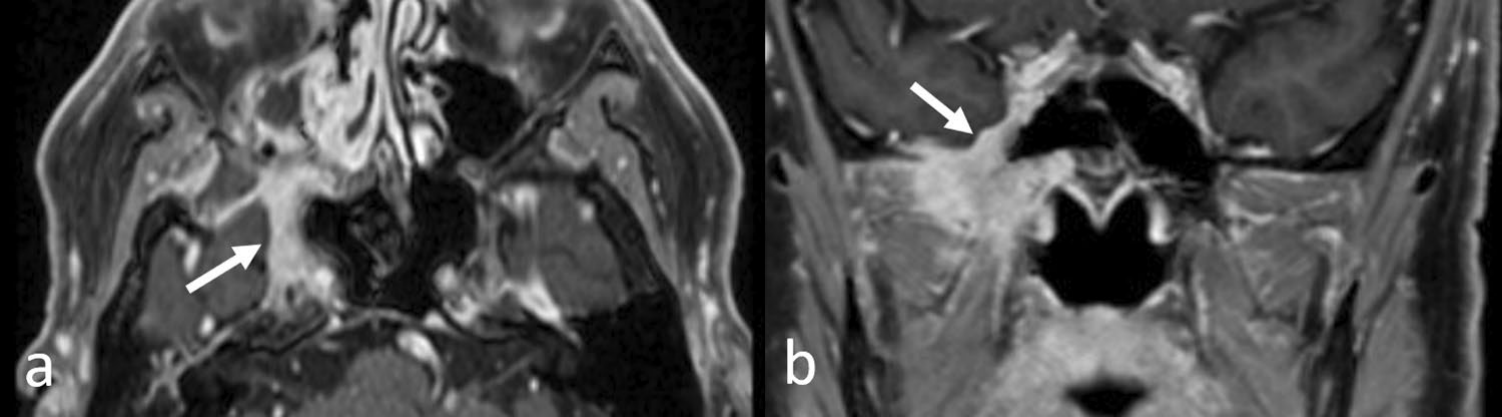
Perineural spread of cranial nerve V in a patient with a sphenoidal squamous cell carcinoma. Axial **a** and coronal **b** reconstructed contrast-enhanced 3D fat-suppressed T1-weighted images show enlarged and enhanced cranial nerve V with its characteristic location (arrows)

PNS is defined as an extension of malignant tumors along the neural sheath [56], and while any CN and its branches can be involved by the PNS, CN V and VII are the most commonly affected. PNS may occur in the absence of hematogenous or lymphatic metastasis and remains clinically unrecognized in some cases [56]. Importantly, up to 40% of patients with radiographically diagnosed PNS are asymptomatic [56]. On head MRI, i.e., CE-T1WI with FS, especially CE-3DT1WI with FS using MPR, enhanced conspicuity of the enlarged, uniformly bright nerve based on their characteristic locations are seen (Fig. 18) [57, 58]. In patients with PNS in CN V, the tumor spreads into the Meckel cave and replaces the normal hyperintense CSF with isointense soft tissue on T2WI [57].

### Double cancers

**Fig. 19:**
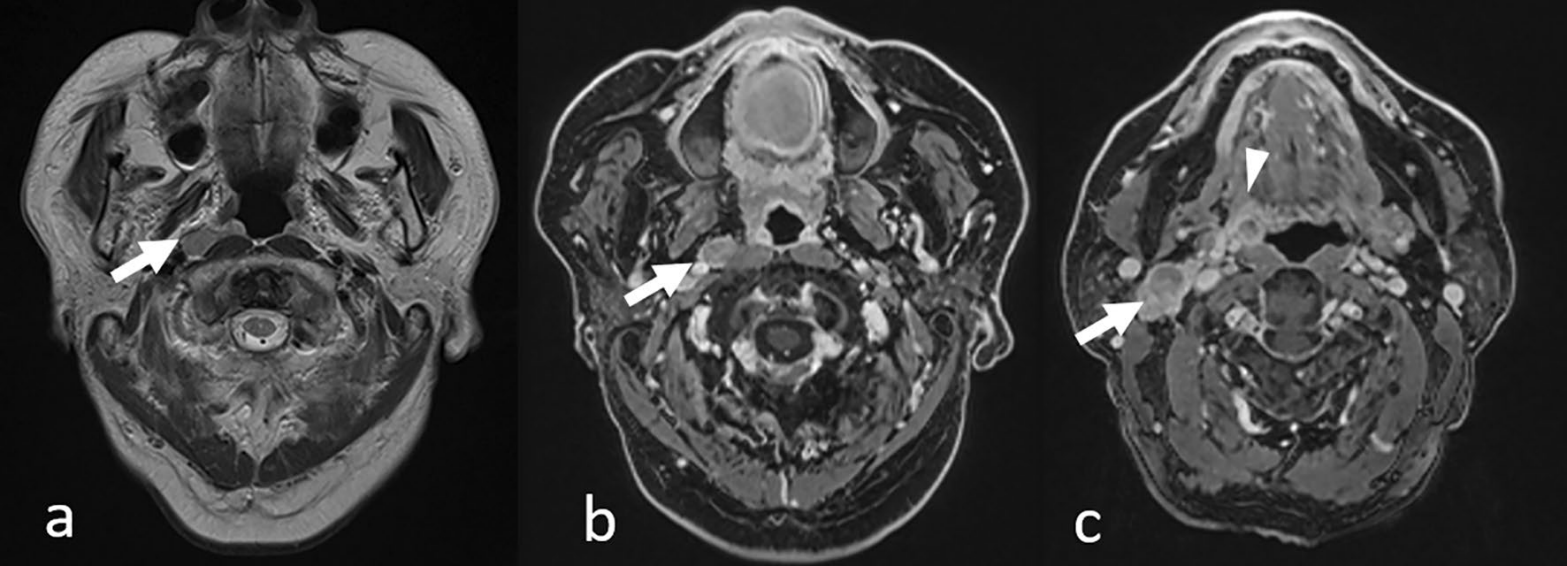
Incidentally detected mesopharyngeal carcinoma (p16 negative) with lymph node metastases in a patient with lung adenocarcinoma. T2-weighted image **a** shows right retropharyngeal lymph node metastasis as a hyperintense nodule in the retropharyngeal area (arrow). Contrast-enhanced 3D T1-weighted images with fat suppression of axial reconstruction (**b** and **c**) show an enlarged right retropharyngeal lymph node (b, arrow), right superior internal jugular nodule (c, arrow), and right mesopharyngeal tumor (c, arrowhead)

When interpreting head MRI scans, attention should also be paid to the head and neck areas, especially in patients with metastatic lesions from lung cancer, as some cancers share common risk factors and double cancers may be observed. For example, smoking is a risk factor for many cancers, including most lung, and head and neck cancers. On head MRI, head and neck cancers and/or lymph node metastases can be incidentally detected (Fig. 19). Retropharyngeal lymph node metastases, which are not easily noted clinically, are well-delineated on head MRI. Attention should be paid, especially in the lower areas of the imaging range.

## Conclusions

A “brain MRI” is a “head MRI.” Nonbrain lesions, including metastases, invasions, and double cancers, can be incidentally detected by head MRI for metastatic brain tumor screening. When interpreting head MRI scans, attention must be paid to not only the intracranial area but also to the extracranial regions. The use of reading checklists and MPR images of CE-3DT1WI with FS can help avoid overlooking nonbrain lesions.
